# Modulation of Aβ_42 _low-n oligomerization using a novel yeast reporter system

**DOI:** 10.1186/1741-7007-4-32

**Published:** 2006-09-26

**Authors:** Sviatoslav Bagriantsev, Susan Liebman

**Affiliations:** 1 University of Illinois, Department of Biological Sciences, 900 S Ashland Ave., Rm. 4068, Chicago, Illinois, 60607, USA

## Abstract

**Background:**

While traditional models of Alzheimer's disease focused on large fibrillar deposits of the Aβ_42 _amyloid peptide in the brain, recent work suggests that the major pathogenic effects may be attributed to SDS-stable oligomers of Aβ_42_. These Aβ_42 _oligomers represent a rational target for therapeutic intervention, yet factors governing their assembly are poorly understood.

**Results:**

We describe a new yeast model system focused on the initial stages of Aβ_42 _oligomerization. We show that the activity of a fusion of Aβ_42 _to a reporter protein is compromised in yeast by the formation of SDS-stable low-n oligomers. These oligomers are reminiscent of the low-n oligomers formed by the Aβ_42 _peptide *in vitro*, in mammalian cell culture, and in the human brain. Point mutations previously shown to inhibit Aβ_42 _aggregation *in vitro*, were made in the Aβ_42 _portion of the fusion protein. These mutations both inhibited oligomerization and restored activity to the fusion protein. Using this model system, we found that oligomerization of the fusion protein is stimulated by millimolar concentrations of the yeast prion curing agent guanidine. Surprisingly, deletion of the chaperone Hsp104 (a known target for guanidine) inhibited oligomerization of the fusion protein. Furthermore, we demonstrate that Hsp104 interacts with the Aβ_42_-fusion protein and appears to protect it from disaggregation and degradation.

**Conclusion:**

Previous models of Alzheimer's disease focused on unravelling compounds that inhibit fibrillization of Aβ_42_, i.e. the last step of Aβ_42 _assembly. However, inhibition of fibrillization may lead to the accumulation of toxic oligomers of Aβ_42_. The model described here can be used to search for and test proteinacious or chemical compounds for their ability to interfere with the initial steps of Aβ_42 _oligomerization. Our findings suggest that yeast contain guanidine-sensitive factor(s) that reduce the amount of low-n oligomers of Aβ_42_. As many yeast proteins have human homologs, identification of these factors may help to uncover homologous proteins that affect Aβ_42 _oligomerization in mammals.

## Background

Alzheimer's disease (AD) is a severe neurodegenerative disorder characterized by an extracellular deposition of amyloid plaques, and an intraneuronal accumulation of neurofibrillary tangles in the brain of affected individuals. A 42 amino acid long Aβ_42 _peptide generated by proteolytic processing of the APP protein is a major component of the amyloid plaques, in which it is mainly represented in the form of detergent-insoluble amyloid fibers (reviewed in [1]). Historically, the Aβ_42 _fibers have been considered to be the major pathogenic agents of AD. Recently, this hypothesis has been challenged by findings suggesting that fibrillar aggregates may represent inert dead-end products of the Aβ_42 _aggregation pathway. Considerable evidence now suggests that the primary neurotoxic effects are associated with soluble SDS-stable assemblies of Aβ_42_, such as 56 kDa Aβ_42 _dodecamers [2], or even smaller, low-n (dimers, trimers, and tetramers) oligomers of Aβ_42_, which seem to appear during the early stages of Aβ_42 _assembly (reviewed in [1,3-5]), and could give rise to larger oligomers. Thus, the focus of putative therapeutic interventions have shifted towards unraveling compounds that inhibit the earliest stages of Aβ_42 _oligomerization. A number of chemical screens have uncovered molecules that inhibit fibrillization of the Aβ_42 _peptide (reviewed in [6,7]). An interesting *Escherichia coli *model of protein solubility control was recently suggested by M. Delisa and colleagues [8], which the authors used to isolate solubility-enhanced variants of Aβ_42_. These studies, however, did not directly address the issue of inhibiting the earliest stages of Aβ_42 _assembly, i.e. formation of the SDS-stable soluble low-n oligomers. This aspect is important, as inhibition of the wrong step may lead to accumulation of toxic Aβ_42 _intermediates.

Yeast *Saccharomyces cerevisiae *is a simple and readily manipulable organism that has been successfully used as a model for various medicinal studies (reviewed in [9,10]), including neurodegenerative disorders, associated with the deposition of amyloid aggregates [11-18]. One of the most valuable contributions of yeast biology to the investigation of neurodegenerative disorders in animals was made by studying yeast prions (reviewed in [19-21]). The yeast translational termination factor Sup35p can form self-propagating infectious amyloid aggregates that arise spontaneously in the cell and manifest a prion phenotype referred to as [*PSI*^+^]. The essential Sup35p protein is composed of three domains. The 124 amino acid long N-terminal domain (N) is glutamine and aparagine rich, dispensable for viability, and required and sufficient for the prion properties of Sup35p. While the function of the highly charged middle (M) domain remains unclear, the C-terminal RF (release factor) domain of Sup35p performs termination of protein translation and is essential for viability.

Prion aggregates of Sup35p are transmitted to daughter cells along with the cytoplasm from the mother cell during cell division [22]. The yeast chaperone Hsp104, a member of the AAA+ protein family [23,24] is required for the successful maintenance of the [*PSI*^+^] prion [25]. Hsp104 shears the SDS-stable Sup35p prion amyloid aggregates into smaller structures in an ATP-dependent manner [26,27] and therefore maintains them in numbers sufficient for the successful transmission to the daughter cell [28,29]. The ATPase activity of Hsp104 is inhibited by millimolar concentrations of guanidine [30], which is therefore employed as a yeast prion-curing agent [31].

In this study, we describe a yeast model of the initial steps of Aβ_42 _oligomerization. We show that the easily scored activity of Sup35p's MRF domain is impaired in AβMRF fusions because the Aβ_42 _causes the fusion to form SDS-stable low-n oligomers. Furthermore, we found that guanidine treatment increases, while gene disruption of Hsp104 decreases, oligomerization of the fusion protein, and that these changes are reflected by the yeast phenotype. This model system represents a convenient tool to perform chemical and genetic screens for agents that interfere with the earliest steps of Aβ_42 _oligomerization.

## Results and discussion

**Figure 1:**
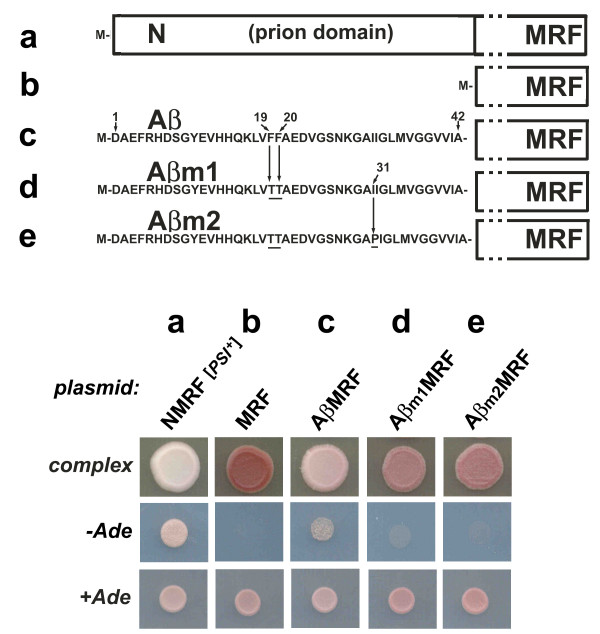
**AβMRF causes nonsense suppression in yeast**. (Upper panel) Schematic illustration of the constructs used in this study: **(a) **full length Sup35p (NMRF) **(b) **Sup35p without the N-terminal prion domain (MRF) **(c) **Aβ_42 _fused to the N terminus of MRF (AβMRF) **(d) **AβMRF carrying a double mutation of Phe19,20Thr in its Aβ_42 _portion (Aβm1MRF) **(e) **AβMRF carrying a triple mutation of Phe19,20Thr and Ile31Pro in its Aβ_42 _portion (Aβm2MRF). All these constructs carry an HA tag between the M and RF domains. Images shown are not to scale. (*Lower panel*) Equal numbers of *ade1-14 *cells containing a genomic deletion of *SUP35 *(*sup35*Δ), and carrying the indicated constructs (**a-e**) on a plasmid were grown on complex medium, or synthetic medium supplemented (+Ade) or not (-Ade) with adenine. (**a**) Cells with inactivated NMRF ([*PSI*+]) had an impaired translational termination activity, were white and grew on -Ade. (**b**) Cells with fully active MRF (lacking the aggregation-prone prion, N, domain), were red and failed to grow on -Ade. (**c**) Cells expressing AβMRF have an impaired translational termination activity, as they were white and grew on -Ade. (**d, e**) The translational termination activity was restored by F19,20T (Aβm1MRF) and F19,20T/I31P (Aβm2MRF) mutations in the Aβ42 region of the fusion protein, making the cells dark pink and preventing their growth on -Ade.

The activity of the essential translational termination factor Sup35p (NMRF) is conveniently assayed *in vivo *by examining the efficiency with which protein synthesis terminates at a premature stop codon (a nonsense-suppression assay, for review see [2,33]; Fig. 1). The assay uses the *ade1-14 *nonsense allele. Strains carrying this mutation and bearing fully active NMRF produce only a truncated (inactive) version of Ade1p, and as a result cannot grow on synthetic medium lacking adenine (-Ade), while they grow normally on synthetic medium supplemented with adenine (+Ade). In addition, these cells accumulate a red intermediate of the adenine synthesis pathway when grown on complex medium. However, if the efficiency of translational termination at the premature stop codon of the *ade1-14 *allele is compromised, the cells gain the ability to grow on -Ade (i.e. they become Ade^+^) and do not accumulate red pigment. For example, cells expressing the complete Sup35p containing the N (prion), M (middle), and RF (release factor) domain, are white and Ade^+ ^when NMRF is in the aggregated [*PSI*^+^] prion form (Fig. 1a). Cells expressing an aggregation-deficient and therefore fully functional form of Sup35p lacking the non-essential N-terminal domain (MRF) are red and Ade^- ^(Fig, 1b). Thus, this well established system reliably distinguishes between fully active monomer, and malfunctioning aggregated forms of NMRF [19,25,34].

To establish a model of Aβ_42 _oligomerization in *S. cerevisiae*, we fused the Aβ_42 _peptide with MRF (Sup35p lacking the N-terminal domain), and containing an HA tag between the M and RF domains. The resulting protein, AβMRF (see upper panel of Fig. 1 for constructs used in this study) was mutagenized in its Aβ_42 _portion according to a recent model of Aβ_42 _oligomerization [35]. The model suggests that binding of one Aβ_42 _molecule to another occurs through four regions: amino acids 15–21, 24–32, 35–37, and 40–42 of one molecule bind to the corresponding regions in another molecule. Substitutions of Phe19, Phe20, and Ile31 were previously shown to inhibit aggregation of Aβ_42 _*in vitro *and prevent its neurotoxic effects [35-37]. To obtain an oligomerization-deficient control for the AβMRF fusion protein, we disrupted the first and the second aggregation-important regions of Aβ_42 _by making double Aβ^F19,20T^MRF (Aβm1MRF) or triple Aβ^F19,20T/I31P^MRF (Aβm2MRF) substitutions in the Aβ_42 _portion of AβMRF. These constructs were expressed in a 74D-694 (*ade1-14*) *sup35*Δ strain, and therefore were the only sources of the essential Sup35p's RF domain.

Yeast cells expressing the AβMRF fusion protein were white on complex medium and grew on -Ade, suggesting that the translation termination activity of the fusion protein was impaired (Fig. 1). In contrast, yeast expressing Aβm1MRF or Aβm2MRF were dark pink on complex medium and Ade^-^, suggesting that the efficiency of translation termination was almost completely restored by the mutations in the Aβ_42 _portion of the fusion protein. No growth difference was detected in the control experiment on +Ade medium. These results are consistent with the hypothesis that the presence of Aβ_42 _in the fusion protein caused it to aggregate into SDS-stable oligomers, thereby affecting its translation termination activity.

**Figure 2:**
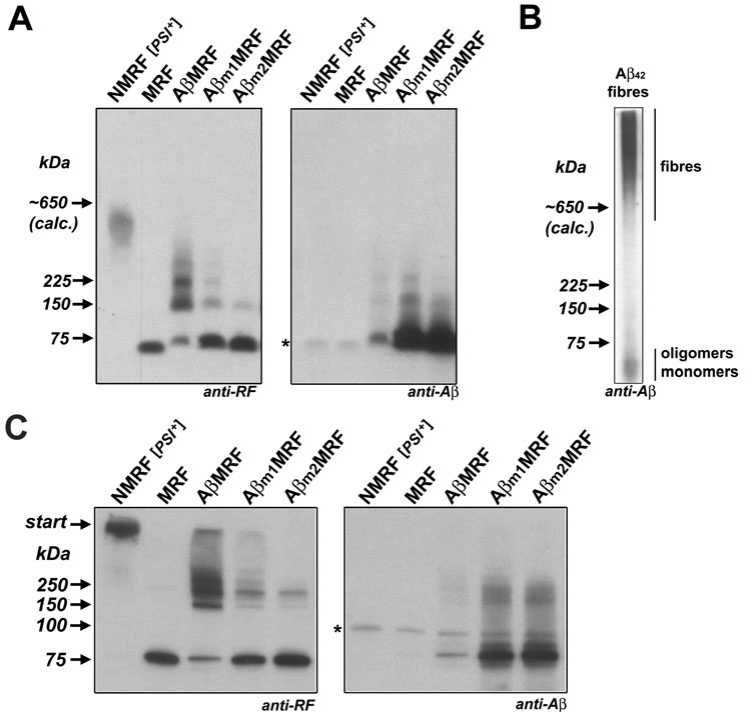
**AβMRF forms SDS-stable oligomers in yeast**. **(A) **Immunoblot analysis of lysates from *sup35*Δ cells containing prionized NMRF ([*PSI*+]) or other indicated constructs. Lysates were treated with 1% SDS for 7 mins at room temperature and resolved by electrophoresis in agarose. Immunoblot analysis was performed using anti-RF antibodies, followed by stripping and staining with anti-Aβ antibodies. The positions of molecular weight standards, treated identically to the experimental samples, are shown (calc., calculated position). AβMRF formed SDS-stable low-n oligomers that largely disappeared after the introduction of the F19,20T (Aβm1MRF) and F19,20T/I31P (Aβm2MRF) mutations into the Aβ_42 _portion of the fusion protein. The decreased efficacy with which anti-Aβ antibodies recognized oligomers of AβMRF suggests that oligomerization occurred through the Aβ_42 _portion of the fusion protein. **(B) **5 mg of amyloid fibers of Aβ_42 _peptide were treated with 1% SDS, resolved in agarose and analyzed by immunoblotting with anti-Aβ antibodies. Only a fraction of Aβ42 fibres can enter the 1.5% agarose gel. **(C) **Same as in **(A) **but the samples were resolved in an acrylamide gel. Asterisk denotes non-specific antibody interaction.

To test whether AβMRF formed SDS-stable oligomers, we analyzed yeast lysates treated with 1% SDS at room temperature by immunoblotting. As shown elsewhere [26], prionized NMRF ([*PSI*^+^]) migrates in the form of SDS-stable aggregates, while MRF, which is unable to prionize, is monomeric (Fig. 2). The pool of AβMRF contained both monomers and SDS-stable complexes migrating at the predicted positions for AβMRF low-n oligomers (dimers, trimers, and tetramers) (Fig. 2). In agarose gels, the AβMRF monomers (calculated molecular weight ~73.7 kDa) migrated at ~65 kDa (Fig. 2A), rather than at ~77 kDa as they did in the acrylamide gels (Fig. 2C). Nevertheless, the positions of the SDS-stable complexes increased with monomer size increments in both gel systems. The SDS-stable oligomers of AβMRF were able to withstand treatment with 2% SDS at room temperature and disaggregated into monomers only after boiling (not shown). We hypothesize that the presence of Aβ_42 _confers AβMRF with the ability to form low-n oligomers (dimers, trimers, and tetramers) similar to the oligomerization of the Aβ_42 _peptide *in vitro *and in the human brain [38-41].

**Figure 3:**
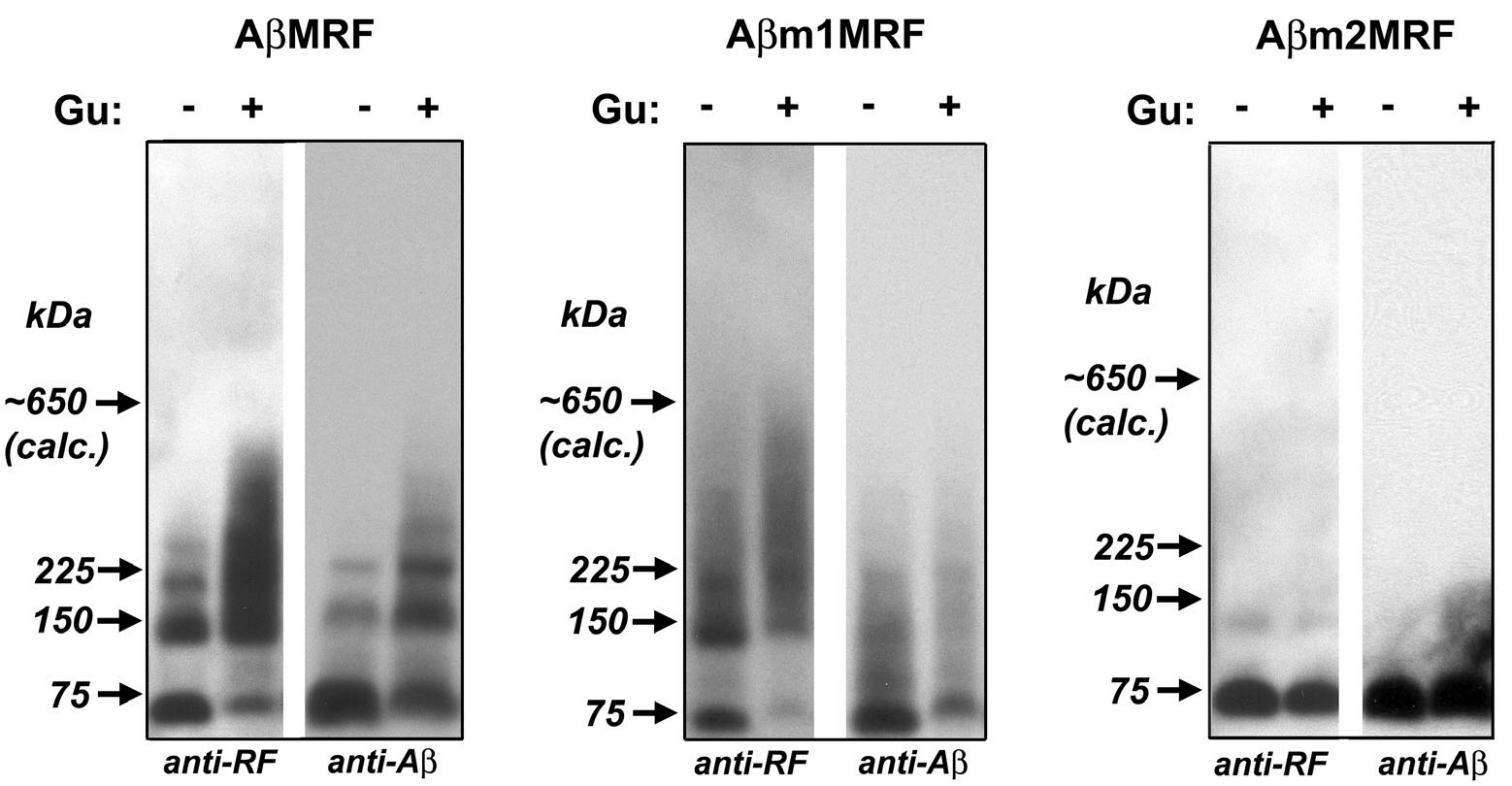
**Guanidine stimulates oligomerization of AβMRF**. *sup35*Δ cells expressing the indicated constructs were grown in the absence (-) or presence (+) of 6.3 mM guanidine (Gu). Equal amounts of lysate proteins were treated with 1% SDS and analyzed by immunoblotting with anti-RF or anti-Aβ antibodies following electrophoresis in agarose. Equal protein loading on each panel was confirmed by coomassie staining of the membrane (not shown).

Unlike antibodies against the RF domain, the efficacy with which anti-Aβ antibodies recognized oligomers of AβMRF decreased as the size of the oligomers increased (Fig. 2A,C, compare left and right panels). Nevertheless, oligomers of AβMRF were stably detected by these antibodies (Fig. 3, left panel). We observed a similar phenomenon when we detected SDS-stable amyloid oligomers of NMRF, which represent structures interacting through the ~14 kDa N-terminal domains, while the ~70 kDa MRF domains on their C termini are exposed to the solvent. The efficiency with which these oligomers are detected with anti-N terminal antibodies is lower that with antibodies against the C-terminal RF domain [42,43]. Apparently, even after SDS electrophoresis the physical access of the antibodies to the target epitopes remains impeded due to the assembly state of the target protein. These observations further corroborate our hypothesis that AβMRF molecules are oligomerized through their Aβ_42 _portions.

To obtain additional evidence that it is the intact Aβ_42 _peptide fused to the MRF that conferred the AβMRF fusion protein with the ability to form low-n oligomers, we analyzed point mutants of AβMRF by SDS-electrophoresis and immunoblotting. We expected that mutations in the aggregation-important regions of Aβ_42 _would inhibit oligomerization of the fusion protein. Consistent with our expectations, disruption of a single aggregation-important region of Aβ_42 _(Aβm1MRF) reduced its ability to form low-n oligomers (Fig. 2). Disruption of a second aggregation-important region (Aβm2MRF) further inhibited oligomerization of the protein. However, small amounts of Aβm2MRF were found in the form of dimers. This is probably due to the fact that this construct still retained two out of four aggregation-important regions intact. The presence of Aβm2MRF dimers explains our observation that these cells were dark pink on complex medium, and were not entirely as red as when a non-tagged MRF protein was expressed (Fig. 2). In addition, the mere presence of Aβ_42 _on the N-terminus of MRF might slightly inhibit the translation termination activity of the fusion protein. Nevertheless, the fact that point mutations in the aggregation-important regions of Aβ_42 _impeded oligomerization of AβMRF and restored its activity, illustrates the ability of the system to clearly distinguish between different levels of AβMRF oligomerization.

We did not detect AβMRF oligomers using the generic oligomer-specific antibodies that recognize oligomers of different amyloidogenic proteins ([44], and data not shown). These antibodies appear not to recognize oligomers of Aβ_42 _smaller than octamers [44], and the oligomers of AβMRF did not reach this size. Nor could we detect AβMRF-containing structures that correspond to fibres of AβMRF, although we successfully used SDS-electrophoresis in agarose previously to analyze different amyloid fibres [43]. In Figure 2B we show that amyloid fibres made of recombinant Aβ_42 _peptide are detected in the upper part of the agarose gel, while AβMRF never formed structures of this size (Fig. 2A). It is possible that the absence of large AβMRF assemblies can be attributed to disaggregating activity of unknown cellular factors, or that the presence of a large MRF domain impedes the ability of AβMRF to assemble into structures larger than the low-n oligomers.

**Figure 4:**
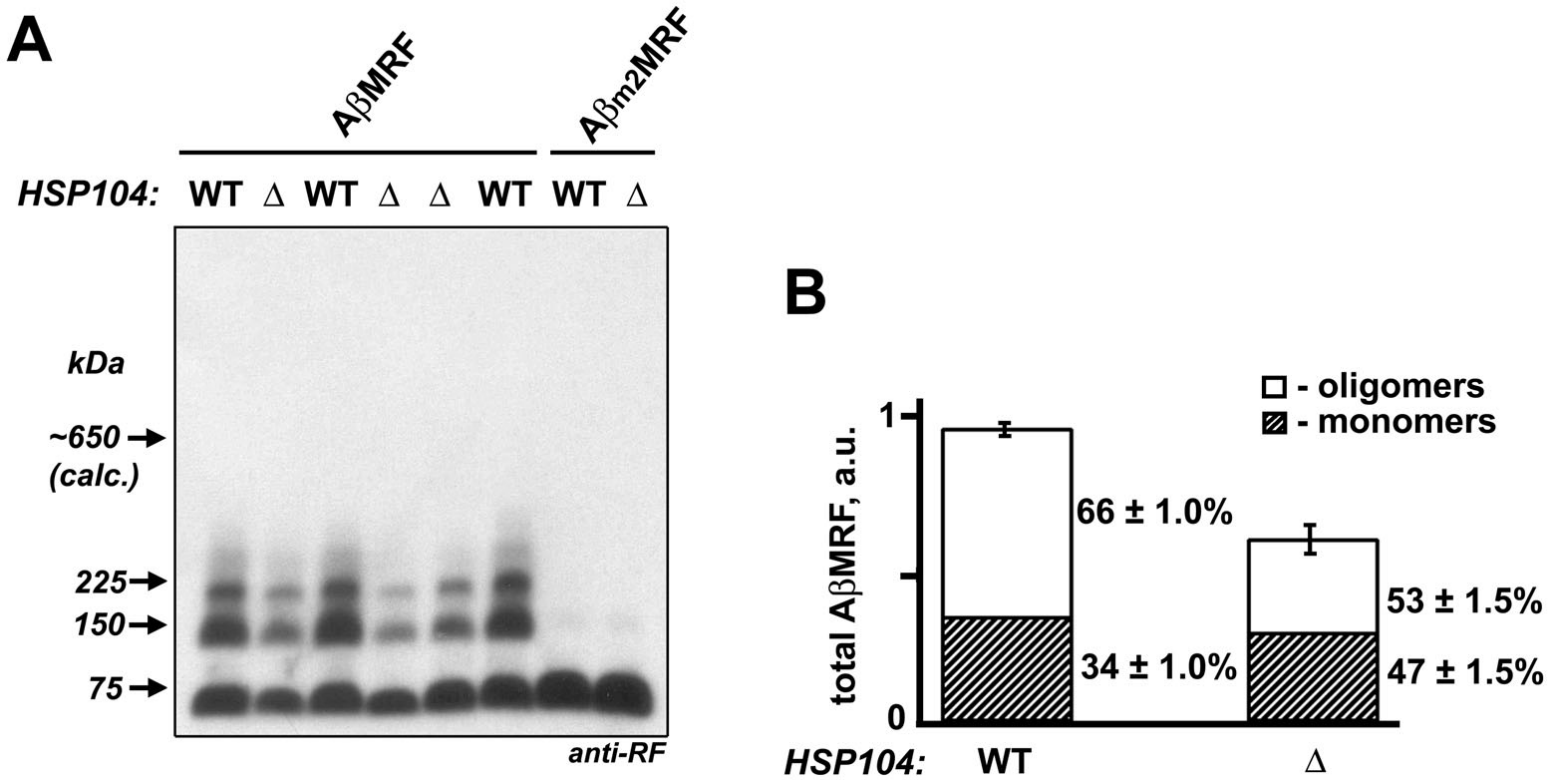
**Deletion of *HSP104 *decreases the total amount of AβMRF and reduces the proportion of oligomers**. **(A) **AβMRF or Aβm2MRF were expressed in a *sup35*Δ strain in the presence (WT) or absence (Δ) of *HSP104*. Equal amounts of lysate proteins were treated with 1% SDS and analyzed by immunoblotting with anti-RF antibodies following electrophoresis in agarose. Equal protein loading was confirmed by coomassie staining of the membrane (not shown). **(B) **The effects of *HSP104 *deletion on the total amount of AβMRF and the ratio between oligomers and monomers from panel A were evaluated by densitometry. The height of the bars reflects total amount of AβMRF relative to that in *HSP104 *WT cells (error bars: s.e., n = 3). Each bar is subdivided according to the content of oligomers (open) and monomers (shaded) of AβMRF (± s.e., n = 3). The deletion of *HSP104 *decreased the total amount of AβMRF and decreased the ratio of oligomers to monomers.

**Figure 5:**
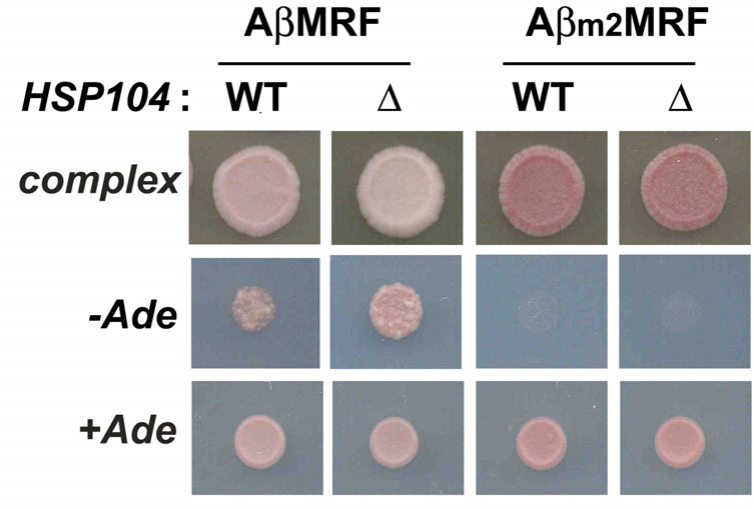
**Deletion of *HSP104 *exacerbates the translation termination defect of AβMRF**. Equal numbers of *sup35*Δ yeast containing (WT) or lacking (Δ) *HSP104 *and expressing AβMRF or Aβm2MRF were grown on complex medium, or synthetic medium supplemented (+Ade) or not (-Ade) with adenine. Deletion of *HSP104 *stimulated growth of AβMRF-expressing cells on -Ade, while having no effect on yeast grown on +Ade medium.

As growth of [*PSI*^+^] yeast in the presence of the prion curing agent guanidine [31] increased the size of SDS-resistant Sup35p aggregates [26], we wondered if it would affect AβMRF oligomers similarly. Thus we grew *sup35*Δ cells expressing AβMRF, Aβm1MRF, or Aβm2MRF in the presence of 6.3 mM guanidine. Strikingly, guanidine dramatically increased the amount of AβMRF and Aβm1MRF oligomers, while depleting the monomeric pool of the proteins (Fig. 3). No such effect was detected with the control Aβm2MRF protein. As guanidine enhances the Sup35p [*PSI*^+^] aggregate size by inhibiting the ATPase activity of Hsp104, we wondered if guanidine's effect on the accumulation of AβMRF oligomers was likewise due to Hsp104 inhibition. Contrary to this hypothesis, deletion of *HSP104 *decreased the proportion of AβMRF oligomers in three independent *hsp104*Δ clones (Fig. 4). In addition, *hsp104*Δ led to a decrease in the total amount of AβMRF in the cells by ~40%, while having no effect on the level of Aβm2MRF protein (Fig. 4). As might be expected, such a decrease in the total amount of AβMRF in *hsp104*Δ cells resulted in more frequent readthrough of the premature stop codon of the *ade1-14 *allele: *hsp104*Δ caused AβMRF-expressing *sup35*Δ cells to grow slightly better on -Ade, while having no effect on Aβm2MRF-expressing *sup35*Δ cells (Fig. 5).

**Figure 6:**
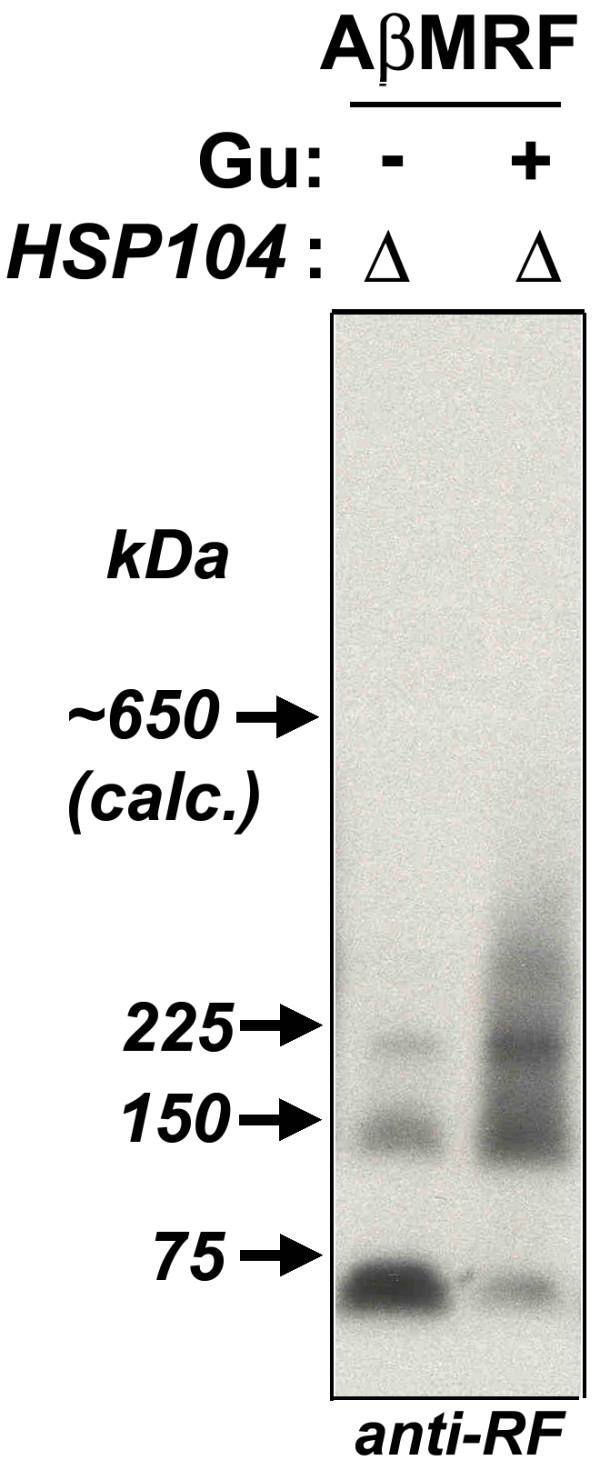
**Guanidine stimulates oligomerization of AβMRF in the absence of *HSP104***. AβMRF-expressing *sup35*Δ *hsp104*Δ cells were grown in the absence (-) or presence (+) of 6.3 mM guanidine (Gu). Equal amounts of lysate proteins were treated with 1% SDS and analyzed by immunoblotting with anti-RF antibodies following electrophoresis in agarose. Equal protein loading was confirmed by coomassie staining of the membrane (not shown).

These results suggest that Hsp104 is not the target of guanidine that stimulated AβMRF oligomerization, and that guanidine therefore may affect other cellular factors. Corroboratively, guanidine stimulated oligomerization of AβMRF even in the absence of *HSP104 *(Fig. 6).

**Figure 7:**
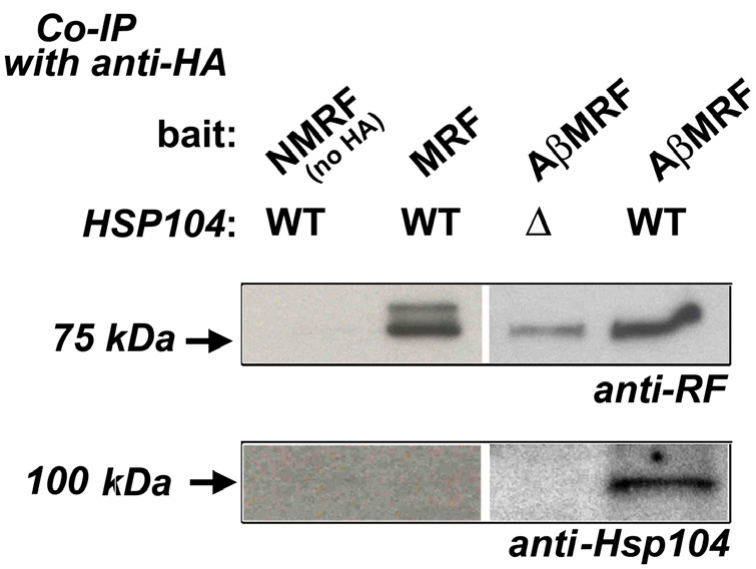
**Co-immunoprecipitation of Hsp104 with AβMRF**. Lysates of *sup35*Δ cells with (WT) or without (Δ) *HSP104*, expressing non-tagged NMRF, HA-tagged MRF, or HA-tagged AβMRF, were incubated with anti-HA antibodies immobilized on agarose beads. Co-precipitated proteins were eluted and analyzed by immunoblotting with anti-RF and anti-Hsp104 antibodies. Hsp104 co-immunoprecipitated with AβMRF, but not with MRF. Non-HA-tagged NMRF was used as a control for non-specific binding to anti-HA antibodies.

To test if Hsp104 interacts with AβMRF, we used HA-tagged AβMRF and HA-tagged MRF to co-immunoprecipitate Hsp104. Indeed, Hsp104 co-immunoprecipitated with AβMRF, but not with MRF (Fig. 7). This is consistent with observations made elsewhere [45] suggesting that Hsp104 interacts with Aβ_42 _*in vitro*. We hypothesize that in our system oligomers of AβMRF undergo continuous disaggregation, and at the same time oligomers and monomers of AβMRF undergo degradation, as a result of interaction between an unknown cellular factor(s) and the Aβ_42 _portion of AβMRF. We show that Hsp104 binds to the Aβ_42 _portion of AβMRF and may therefore physically impede interaction between Aβ_42 _and the factors that trigger degradation and disaggregation of the fusion protein. Consistent with this, deletion of *HSP104 *led to a ~40% decrease in the total amount of the AβMRF protein (Fig. 4), possibly as a result of increased susceptibility of AβMRF to degradation-triggering factors. At the same time, deletion of *HSP104 *shifted the equilibrium between oligomers and monomers such that the monomer's share in the overall pool of AβMRF increased from 34 to 47%, possibly as a result of AβMRF disaggregation. As disaggregation of protein aggregates in yeast usually requires energy from ATP [46-48], it is tempting to speculate that guanidine may specifically inhibit the ATPase activity of the unknown disaggregating factors, as guanidine is able to inhibit the ATPase activity of Hsp104 [30].

Recent evidence suggests that chaperones play critical roles in protecting neuronal cells from the deleterious effects of amyloid aggregates and their precursors (reviewed in[49]). Such a protective mechanism may involve degradation and/or disaggregation of toxic intermediates. In yeast, disaggregation of aggregated protein is carried out by the chaperone machinery, which includes Hsp104, Hsp70/Hsp40, and small heat shock proteins (sHsp) Hsp42, and Hsp26 [23,46,50,51]. All of these chaperones except for Hsp104 have homologs in mammals. It was shown that Hsp26 facilitates disaggregation and refolding of thermally denatured firefly luciferase [47] and citrate synthase [48] by Hsp104 and Hsp70/Hsp40. The disaggregating activity of the yeast chaperone machinery is not limited to amorphous protein aggregates. Overexpression of Hsp104 together with Hsp26 and Hsp42 [47], or Hsp70 together with Hsp40 [52], or Hsp70 alone [18] increased the solubility of polyglutamine aggregates in yeast models of Huntington's disease, while deletion of *HSP104 *led to solubulization of polyglutamine aggregates [53]. The direct implication of chaperone machinery to the pathology of Alzheimer's disease is still obscure. The yeast system described in this study provides an opportunity to examine the ability of different compounds (proteinacious or chemical) to interfere with the process of AβMRF oligomerization. Abrogation of oligomerization of the AβMRF will lead in our system to the accumulation of AβMRF monomers, causing inhibition of growth in the absence of adenine, and a redder color on complex medium, thus providing a simple functional readout.

## Conclusion

In this study, we present a yeast model system focused on the initial steps of Aβ_42 _oligomerization. We fused the Aβ_42 _peptide to the MRF domain of the yeast translation termination factor, Sup35p, and monitored its activity by the growth of yeast on different media. The presence of the Aβ_42 _caused the AβMRF fusion protein to form SDS-stable low-n oligomers, which appear to mimic the ability of the natural Aβ_42 _peptide to form low-n oligomers. The oligomerization of AβMRF compromised its translational termination activity causing a more frequent readthrough of the *ade1-14*'s premature stop codon, which was easily scored by yeast growth (Fig. 1). Point mutations previously shown to inhibit Aβ_42 _aggregation *in vitro*, were made in the Aβ_42 _portion of the fusion protein. These mutations both inhibited oligomerization and restored activity to the fusion protein. Thus, using this reporter system it is possible to assay the degree of AβMRF oligomerization by examining the growth of yeast on complex and adenine-deficient media. We also demonstrate that the yeast prion curing agent guanidine enhances the level of SDS-stable AβMRF oligomers, presumably by inactivating factors that degrade and/or disaggregate them. This effect is not caused by inactivation of the yeast chaperone Hsp104, which appears to protect AβMRF from the effects of such factors. This model system represents a convenient tool to test or perform chemical and genetic screens for agents that interfere with the earliest steps of Aβ_42 _oligomerization.

## Methods

### Yeast strains and media

Derivatives of yeast strain 74D-694 (*MATa ade1-14 ura3-52 leu2-3,112 trp1-289 his3-200 *[54]) containing a genomic deletion of *SUP35 *(*sup35*Δ::*LEU2*), or a double deletion of *SUP35 *and *HSP104 *(*hsp104*Δ::*URA3*) [55] (kind gifts from Drs. C. G. Crist and Y. Nakamura) were used in this study. Since the RF domain of Sup35p is essential, viability of the *sup35*Δ 74D-694 (L2723) and *hsp104*Δ*sup35*Δ 74D-694 (L2725) strains was maintained by a pRS313-based (*CEN*, *HIS3*) plasmid encoding full length Sup35p [56]. A [*PSI*^+^] derivative of the *sup35*Δ 74D-694 strain was described earlier [56]. For this study, plasmids encoding full length Sup35p in L2725 and L2723 and were replaced with pRS313 or pRS316-based (*CEN, URA3*) plasmids encoding MRF, AβMRF, or aggregation-deficient derivatives of AβMRF (see below). The absence of the original full length Sup35p was confirmed by immunoblotting with polyclonal antibodies against Sup35p's N domain (Ab0332, a kind gift from Dr. S. Lindquist).

Standard yeast media, cultivation and transformation procedures were used [57]. Yeast was cultivated either in complex medium (YPD: 2% dextrose, 2% bacto peptone, 1% yeast extract), or in complete synthetic medium (an artificial mix of 2% dextrose and all necessary aminoacids and nucleobases) lacking adenine (-Ade), uracil (-Ura), or histidine (-His), as required. Complete synthetic medium was referred in the text as '+Ade' medium. As the RF domain within the fusion proteins is essential, no plasmid selection was required after the strains acquired the desired RF-containing constructs. Expression of the AβMRF constructs driven by the copper-inducible *CUP1 *promoter was stimulated by the addition of 50 μM CuSO_4 _to all media. Where indicated, the media were supplemented with 6.3 mM guanidine hydrochloride.

### Plasmid construction

The pRS316-based *CEN URA3 *plasmid (p1071) encoding full length Sup35p under its native promoter with an HA tag between the M and RF domains, and with the NM domains surrounded by *Bam*HI sites was kindly supplied by Dr. J. Weissman [58]. To construct MRF (HA-tagged Sup35p without the 123 N-terminal amino acids which constitute the prion, N, domain of Sup35p) under its native promoter, a fragment containing the HA-tagged M domain (M^HA^) and a new *Bam*HI site (introduced on primer 1) was PCR amplified from p1071 using primers 1 and 2. The PCR product was cut with *Bam*HI and inserted into p1071 cut with the same enzyme, resulting in p1366, where M replaced NM.

To construct AβMRF under the copper-inducible *CUP1 *promoter (p1364), we PCR amplified a DNA fragment encoding Aβ_42 _flanked by restriction sites, using the overlapping primers 3, and 4, which we designed based on the known aminoacid sequence of the peptide (DAEFRHDSGYEVHHQKLVFFAEDVGSNKGAIIGLMVGGVVIA). The resulting PCR product was cut with *Bam*HI and *Bgl*II and inserted in the correct orientation into p1071 cut with *Bam*HI, yielding p1300, where Aβ_42 _replaced NM. The native *SUP35 *promoter in p1300 was replaced with the *CUP1 *promoter from p984 [59] using *Xho*I and *Bam*HI sites, yielding p1301. A fragment containing M^HA ^and a new *Eco*521 site (introduced on primer *5*) was PCR amplified from p1071 using primers 5 and 6, cut with *Eco*521 and inserted into p1301 cut with the same enzyme in the linker region between Aβ_42 _and RF, resulting in the following construct: *CUP1::met-Aβ*_42_*-HA-M-3xHA-RF *(p1364) referred to herein as AβMRF.

A double substitution in the Aβ_42 _region of AβMRF (Aβ_42 _^F19,20T^MRF, or Aβm1MRF) was introduced into p1364 by site-directed mutagenesis using a Quick-Change (Stratagene) kit, as suggested by the manufacturer, using primers 7 and 8, resulting in p1397. This plasmid was further mutagenized using primers 9 and 10, to obtain Aβ_42 _^F19,20T/I31P^MRF, or Aβm2MRF (p1541).

To shuffle the AβMRF fusions into pRS313 (*CEN*, *HIS3*), corresponding fragments encoding the fusion proteins with their *CUP1 *promoters were cut from p1364, p1397, and p1541 with *Sac*I and *Xho*I and inserted into pRS313 cut with the same enzymes, yielding p1547, p1549 and p1551, respectively.

The expression level and the oligomeric pattern of all corresponding AβMRF fusions expressed from the sibling shuffle vectors pRS313 (*CEN, HIS3*) and pRS316 (*CEN, URA3*) were the same (not shown).

List of primers (5'-3'):

1. GGTTTCCAAGGATCCTCTCAAGGTATGTC

2. CCACCAAACATCCATGGGAATTCTGC

3. AGCTGGATCCATGGATGCAGAATTCCGACATGACTCAGGATATGAAGTTCATCATCAAAAATTGGTGTTCTTTGCAGAAGATGTG

4. ATTAAGATCTCGCTATGACAACACCGCCCACCATGAGTCCAATGATTGCACCTTTGTTTGAACCCACATCTTCTGCAAAGAACAC

5. TCTCACGGCCGGTCTTTGAACGACTTTC

6. CCACCAAACATCCATGGGAATTCTGC

7. CATCATCAAAAATTGGTGACCACTGCAGAAGATGTGG

8. CCACATCTTCTGCAGTGGTCACCAATTTTTGATGATG

9. GGGTTCAAACAAAGGTGCACCAATTGGACTCATGGTGGGCGG

10. CCGCCCACCATGAGTCCAATTGGTGCACCTTTGTTTGAACCC

All plasmids used in this study were analyzed by restriction analysis and sequencing, and their protein products were tested by immunoblot analysis with antibodies against Aβ (6E10), RF (BE4), and HA tag (not shown).

### Yeast growth assay

To compare yeast growth on agar plates, equal numbers of cells (5 μl of cellular suspension with OD_600 _= 2) were spotted on agar plates, and incubated at 30°C for 3 days (complex medum), or 7 days (adenine deficient medium, -Ade). The desirable color saturation on complex medium was achieved by incubating the plates for 3 additional days at 4°C.

### Immunoblotting

To obtain cell lysates, cells grown in 50 ml of liquid medium to late logarithmic stage were pelleted, washed with water, resuspended in a 50 mM Tris pH 7.6 buffer containing 50 mM KCl, 10 mM MgCl_2_, 5% glycerol, 10 mM PMSF, and an anti-protease cocktail for yeast (Sigma) 1:100, and lysed by vortexing with glass beads. Cell debris was removed by centrifugation at 4°C for 5 min at 10,000 ***g***. Protein concentration was measured by the Bradford reagent from BioRad [60].

To visualize SDS-stable oligomers of AβMRF by SDS electrophoresis in polyacrylamide or agarose gels, equal amounts of lysate proteins were treated with sample buffer (50 mM Tris/HCl pH 6.8 for acrylamide, or 25 mM Tris 200 mM glycine for agarose gels, respectively) containing 1% SDS for 7 min at room temperature. Oligomers of AβMRF were also able to withstand 2% SDS treatment at room temperature (not shown). To disaggregate AβMRF oligomers into monomers, lysates were boiled for 5 min in sample buffer supplemented with 2% SDS and 2% β-mercaptoethanol (not shown).

SDS-treated lysates were resolved by SDS-electrophoresis in 7.5% polyacrylamide gels as described [61], and transferred to an Immun-Blot PVDF membrane (Bio-Rad). Immunodetection was performed using monoclonal antibodies against Sup35p's RF domain (BE4, developed by Dr. V. Prapapanich in our laboratory), monoclonal antibodies against Aβ_1–17 _(6E10, from Signet Laboratories), or anti-oligomer antibodies (a kind gift from Drs. R. Kayed and C. Glabe; [44]). Signal was revealed using a Western-Star chemiluminescence development kit (Applied Biosystems) as suggested by the manufacturer. Molecular weight standards were treated in the same sample buffer as the experimental samples, and were revealed after immunodetection by staining the membrane with the Coomassie Brilliant Blue R-250 reagent. The position of the 650 kDa molecular weight marker was calculated using AlphaEaseFC software.

For better resolution of the AβMRF oligomers, we used SDS electrophoresis in agarose as described elsewhere [62], with the following changes. The SDS-treated lysates (see above) were electrophoretically separated in horizontal 1.5% agarose gels in a 25 mM Tris buffer containing 200 mM glycine and 0.1% SDS. Proteins were transferred onto a PVDF membrane in a 25 mM Tris buffer containing 200 mM glycine, 15% methanol, 0.1% SDS using a semi-dry blotting unit FB-SDB-2020 (Fisher Scientific) at 1 mA per cm^2 ^of the gel/membrane surface for 1 hr, and processed as described above. Densitometry was performed using Alpha Imager 2200 (Alpha Innotech) and processed on AlphaEaseFC imaging software.

To confirm equal protein loading, we first determined protein concentrations in the lysates by Coomassie Brilliant Blue (Bradford reagent). We then brought the protein concentration in all samples to the same value, and in the same volume, followed by an additional verification by Bradford reagent. After the immunodetection, the membrane was stained with Coomassie to confirm equal protein loading.

### Aβ_42 _polymerization and immunoblotting

Recombinant Aβ_42 _peptide (powdered Aβ_42_-acetate from Rpeptide) was polymerized according to the manufacturer's suggestions. Briefly, a 1 mg/ml solution of Aβ_42 _was made by resuspending 0.5 mg of Aβ_42 _powder in 100 μl of 2.5 mM NaOH and adding 400 μl of phosphate buffered saline solution. Polymerization proceeded at room temperature with constant rotation (60 rpm). Polymerization was measured by Thioflavin T fluorescence (λ_EX _= 442 nm; λ_EM _= 483 nm). To perform immunoblotting, a sample containing 5 μg of polymerized Aβ_42 _was treated with 1% SDS and resolved by SDS electrophoresis in 1.5% agarose and processed as described above.

### Immunoprecipitation

Samples (500 μl) containing 800 μg of total lysate proteins were incubated with 6 μl of anti-HA antibodies immobilized on agarose beads using a Pro-Found HA-Tag Co-IP kit (Pierce), for 1.5 hrs at 4°C. Following incubation, the beads were washed three times with 0.5 ml of phosphate buffered saline containing 0.05% Tween 20 to remove the non-specifically bound proteins. Immunoprecipitated protein complexes were eluted with hot (95°C) 0.3 M Tris buffer pH 6.8 containing 5% SDS, resolved by electrophoresis in 10% polyacrylamide gels, and analyzed by immunoblotting using monoclonal antibodies against the RF domain or against Hsp104 (SPA-1040, from Stressgen).

## Abbreviations

AD, Alzheimer's disease; Aβ, amyloid-β protein; RF, release factor; SDS, sodium dodecylsulfate; Hsp, heat shock protein; YPD, yeast extract/peptone/dextrose; HA, human influenza hemagglutinin; PCR, polymerase chain reaction; PMSF, phenylmethylsulfonylfluoride; PVDF, polyvinylidenfluoride; Gu, guanidine hydrochloride; WT, wild type; sHsp, small heat shock protein.

## Authors' contributions

S.B. and S.W.L. conceived and designed the experiments, analyzed data, and wrote the manuscript. S.B. performed the experiments and collected data.
